# Salicylic acid improves cowpea productivity under water restriction in the field by modulating metabolism

**DOI:** 10.3389/fpls.2024.1415682

**Published:** 2024-07-02

**Authors:** Igor Eneas Cavalcante, Alberto Soares de Melo, Rener Luciano de Souza Ferraz, Rayanne Silva de Alencar, Guilherme Felix Dias, Priscylla Marques de Oliveira Viana, Maurisrael Moura Rocha, Ashwell Rungano Ndhlala, Francisco Vanies da Silva Sá, Claudivan Feitosa de Lacerda, Pedro Roberto Almeida Viégas

**Affiliations:** ^1^ Posgraduate Program in Agricultural Sciences, State University of Paraiba, Campina Grande, Paraíba, Brazil; ^2^ Empresa Brasileira de Pesquisa Agropecuária, Pesquisa Agropecuária do Meio-Norte, Teresina, Piauí, Brazil; ^3^ Department of Plant Production, University of Limpopo, Sovenga, South Africa; ^4^ Posgraduate Program in Agricultural Engineering, Universidade Federal do Ceará, Fortaleza, Ceará, Brazil; ^5^ Department of Agronomy, Federal University of Sergipe, São Cristóvão, Sergipe, Brazil

**Keywords:** osmoprotection, antioxidant mechanism, photosynthesis, production, *Vigna unguiculata* (L.) Walp.

## Abstract

**Introduction:**

Salicylic acid has shown promise in alleviating water stress in cultivated plants. However, there is a lack of studies confirming its effectiveness in cowpea plants grown in field conditions. Therefore, this research aimed to evaluate the use of salicylic acid as a water stress mitigator in cowpea cultivars under different irrigation depths in field conditions.

**Methods:**

Four cowpea cultivars (BRS Novaera, BRS Tapaihum, BRS Pujante, and BRS Pajeú) were subjected to different treatments: control (W100: 100% replacement of crop evapotranspiration – ETc), W50 (50% of ETc), W50+SA2 (50% of ETc + 276 mg L^-1^ of SA), and W50+SA4 (50% of ETc + 552 mg L^-1^ of SA). The treatments were combined in a 4×4 factorial scheme with three replications, arranged in a randomized block design.

**Results:**

Water restriction had a negative impact on the water status, growth, gas exchange, and production of the cultivars while also leading to changes in the antioxidant metabolism and osmolyte concentration. The application of SA enhanced antioxidant activity and the synthesis of osmotic adjusters under stress conditions. The most effective concentration was 276 mg L^-1^ in stage R2 and 552 mg L^-1^ in stage V7, respectively. The BRS Pujante cultivar showed increased productivity under water restriction with SA application, while the BRS Tapaihum was the most tolerant among the cultivars studied.

**Discussion:**

In summary, our findings underscore the importance of using SA to mitigate the effects of water restriction on cowpea cultivation. These discoveries are crucial for the sustainability of cowpea production in regions susceptible to drought, which can contribute to food security. We further add that the adoption of new agricultural practices can enhance the resilience and productivity of cowpea as an essential and sustainable food source for vulnerable populations in various parts of the world.

## 1 Introduction

Cowpea [*Vigna unguiculata* (L.) Walp.] is a Fabacea widely grown in tropical and subtropical regions as well as is a staple in the diet of many populations due to its high nutritional value (Horn et al., 2022). Its cultivation is a key source of income for rural communities and a major contributor to grain production in these regions (Anago et al., 2021).

In the context, cowpea, being rich in nutrients, is a guarantee of food security, especially for vulnerable populations, in light of the Sustainable Development Goals (SDGs) of the United Nations 2030 Agenda, particularly SDG 2, which aims to eliminate hunger through food security and the promotion of sustainable agriculture (Omomowo and Babalola, 2021).

In Brazil, the cultivation of this fabaceae occurs predominantly in the North and Northeast regions on small and medium-sized rural properties and under rainfed conditions (Guimarães et al., 2020). Thus, despite its rusticity, some research has demonstrated that cowpea productivity in these regions is strongly affected by water deficits in at least one of its phenological phases (Silva et al., 2020; Souza et al., 2020a). According to data published by CONAB (2023), although the northeast region has the largest area cultivated with this species, its productivity (411 kg ha^-1^) is lower than the central-south region (1,024 kg ha^-1^), which presents greater technological innovations.

Water deficiency aggravates the loss of plant productivity by impeding development and restricting the net carbon assimilation. The inhibition of the photosynthetic process is caused by a restriction in stomatal conductance (Souza et al., 2020b), leading to reduced absorption of soil solution, hindered growth and development, and restricted production (Miri et al., 2021; Olorunwa et al., 2021). This restriction also promotes an increase in the concentration of reactive oxygen species (ROS), which can trigger oxidative stress (Khatun et al., 2021).

However, it is important to highlight that plants respond to water restriction by activating a series of mechanisms that help them mitigate the harmful effects of this stress. Osmotic adjustment is characterized by the accumulation of compatible organic solutes in the cell cytosol, which facilitates water absorption from the soil (Santos et al., 2022). Furthermore, the antioxidant enzyme system functions to eliminate ROS and regulate their levels in metabolism (Arif et al., 2023).

Additionally, the application of salicylic acid (SA) can be a promising strategy to mitigate water stress in cowpea plants (Sultana et al., 2024) because it can regulate metabolic pathways and enhance the plants’ defense against water deficits (Ghahremani et al., 2023). The use of silicon has been shown to have positive effects in mitigating the impact of water stress on plants. It promotes an increase in relative water content (Carvalho et al., 2020), the concentration of organic solutes, and the activity of antioxidant enzymes (Shemi et al., 2021; Jales Filho et al., 2023). Silicon also regulates mechanisms that counteract oxidative stress (Arif et al., 2023) and increases the productivity of plants grown under water deficit (El-Sanatawy and Zedan, 2020).

Despite promising results, information regarding the interaction between water replacement levels and SA application in cowpea plants is still scarce under field conditions. Thus, considering its importance for semi-arid regions, such as the Brazilian Northeast, the present study was carried out with the objective of evaluating SA as a water stress attenuator in cowpea cultivars grown under irrigation levels under field conditions.

## 2 Materials and methods

### 2.1 Location and conduct of the experiment

The experiment was carried out in a agricultural area at the Center for Agricultural and Environmental Sciences (CCAA), Campus II of the State University of Paraíba (UEPB), Lagoa Seca-PB. The field conditions were observed from January to April 2020. The geographical coordinates of the location are latitude 7° 09’S, longitude 35° 52’ W, and altitude 634 m. Biochemical analyses were conducted at the Ecophysiology of Cultivated Plants Laboratory (ECOLAB) at UEPB in Campina Grande-PB, Brazil. The laboratory is situated at latitude 07° 13’ 50’’, longitude, 35° 52’ 52’’, and altitude 551 m.

**Figure 1:**
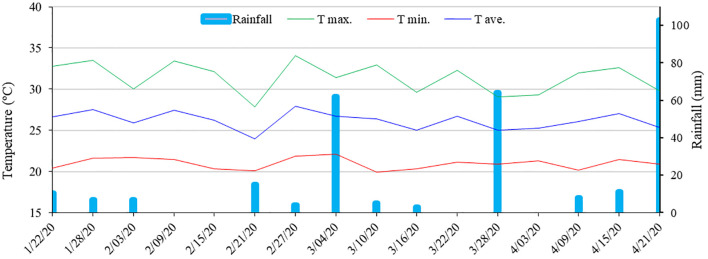
Data on maximum (T max.), minimum (T min.), average (T avg.) temperature (°C) and rainfall (mm) accumulated every six days during the experiment.

The research site has a tropical climate with a dry season, classified as type AS’ by the Köppen system. The annual average temperature is 22°C, with a minimum of 19°C and a maximum of 26°C (**Figure 1**). The average annual precipitation is above 700 mm, with higher rainfall levels concentrated in the months of April to August. The average annual reference evapotranspiration is 500 mm, and the average annual relative humidity is 80%.

### 2.2 Treatments application and experimental design

Four cowpea cultivars (BRS Novaera, BRS Tapaihum, BRS Pujante, and BRS Pajeú) were subjected to control treatments (W100 – plants subjected to 100% replacement of crop evapotranspiration – ETc), W50 (50% of ETc), W50+SA2 (50% of ETc + 276 mg L^-1^ of SA) and W50+SA4 (50% of ETc + 552 mg L^-1^ of SA), combined in a 4×4 factorial design, with three replications. Each experimental unit measured 1.10 m wide and 2.0 m long, with five irrigation lines and 10 plants per line, arranged in a randomized block design. The salicylic acid (P.A. - C_7_H_6_O_3_, molecular weight 138.12 g mol^-1^) was from Sigma-Aldrich.

After applying the fungicide, the seeds were allowed to rest for 24 hours at room temperature and low light. Following this period, all seeds were manually sown, with one seed placed in each hole at a depth of 3.0 cm. The holes were spaced 10 cm apart, and there was a distance of 50 cm between the planting lines (Rocha et al., 2019).

The soil in the experimental area had the following characteristics: sand (86.04%), silt (12.05%), clay (1.91%), soil density (1.62 g cm^-3^), particle density (2.69 g cm^-3^), porosity (39.77%), calcium (2.31 cmol_c_ dm^-3^), magnesium (2.30 cmol_c_ dm^-3^), sodium (0.05 cmol_c_ dm^-3^), potassium (0.27 cmol_c_ dm^-3^), sulfur (4.93 cmol_c_ dm^-3^), hydrogen (0.89 cmol_c_ dm^-3^), aluminum (0.00 cmol_c_ dm^-3^), organic matter (1.10%), and pH (6.62).

Irrigations were applied daily according to crop evapotranspiration (ETc) from 7:00 to 8:00 AM. A drip irrigation system consisting of drip tapes with a wall thickness of 0.2 mm, an internal diameter of 16 mm, and self-compensating emitters with a flow rate of 1.6 L per hour and spaced every 10 cm between emitters and 0.5 m between lines. Water replenishment was calculated using the Penman-Monteith (FAO) method (Allen et al., 1998) based on climate data from the nearby agrometeorological station (7°09’26.1” S, 35°52’16.9W). The reference evapotranspiration (ETo), and total water level (TWL) were determined according to Equations 1, 2 respectively.


(1) $$ET_{0\;}=\frac{0.408\Delta \left(Rn-G\right)+\gamma \frac{900}{T+273}U_{2}\left(e_{s}+e_{a}\right)}{\Delta +\gamma \left(1+0.34U_{2}\right)}$$

Where: ETo = Reference evapotranspiration (mm day^-1^); Rn = net radiation on the culture surface (MJ m^-2^ day^-1^); G = soil heat flux (MJ m^-2^ day^-1^); Δ= slope of the vapor pressure curve versus air temperature kg ha^-1^ (kPa °C^-1^); U2 = wind speed measured at 2.0 m height (m s^-1^); T = temperature (°C); es = water vapor saturation pressure (kPa); ea = real water vapor pressure (kPa); *γ* = psychrometric factor (MJ kg^-1^).


(2) $$\text{TWL}={\text{ET}}_{0}\times \text{Kc}$$

The maximum Kc used for each phenological stage was as follows: initial stage: 15 days (Kc = 0.8); growth stage: 25 days (Kc = 1.1); reproductive stage: 17 days (Kc = 1.4); final stage: 0.3, according to Bastos et al. (2008). The irrigation time was measured after the system reached a stable pressure of 0.8–1.0 kgf cm^-2^. Pressure gauges in the secondary lines were used for measurement. The irrigation level for water deficit (W50) was set at 50% of the irrigation level without water restriction (W100).

Salicylic acid was applied at 18 and 36 days after sowing (DAS - stages V3 and V9, respectively) using 20 mL per plant with a knapsack sprayer at 40 psi pressure, targeting both sides of the leaves (adaxial and abaxial). Irrigation depths for water restrictions were determined the day after the first spraying (19 DAS).

At 10 and 18 DAS, DripSol MAP fertilizer (12% N and 65% P_2_O_5_) was applied through fertigation at a rate of 390 g diluted in 10 L of water per application, using a venturi fertilizer injector. At 37 DAS, 10 mL of Benevia^®^ insecticide (100 g L^-1^, a.i. Ciantraniliprole) was sprayed according to the manufacturer’s recommendations, diluted in 20 L of water, and 4 mL of spreader-sticker. At 18 days after sowing (DAS), weed dry matter was uniformly added to the interrows of experimental area plots to form 5 cm layers (Maia Junior et al., 2019). Manual control of spontaneous plants was carried out throughout the experiment.

At 29 days after SA application (phenological stage V7), leaf gas exchange analyses were carried out using an infrared gas analyzer (IRGA - Infrared Gas Analyzer, GFS 3000 FL). Measurements included net photosynthesis (*A*) (μmol CO_2_ m^-2^ s^-1^) stomatal conductance (*gs*) (mmol m^-2^ s^-1^) and transpiration (*E*) (mmol H_2_O m^-2^ s^-1^) in fully expanded leaves located at the median position of the plant. Three plants from each experimental plot were analyzed. Leaf tissue from one plant per plot was collected to determine free proline (FPR) content using the method by Bates et al. (1973) and total soluble sugars (TSS) content using the sulfuric phenol method by Dubois et al. (1956). The activity of ascorbate peroxidase (APX) and catalase (CAT) enzymes was determined following the methodologies by Nakano and Asada (1981) and Sudhakar et al. (2001), respectively. Results were expressed in nmol of ascorbate min^-1^ g^-1^ of fresh mass (FM) and µmol of H_2_O_2_ min^-1^ g^-1^ of FM.

Leaf water potential (Ψw) was measured using a Scholander-type pressure chamber (Scholander et al., 1965) on one plant per plot. The same chamber was used to calculate the total leaf area (TLA) with ImageJ software. Leaflets were digitized on an HP Deskjet Ink Advantage 2545 Multifunctional Printer at a two-centimeter scale. Total dry mass (TDM) was assessed in plants dried in an oven with air circulation at 70 °C for a period of 48 hours and then weighed on an analytical balance.

**Figure 2:**
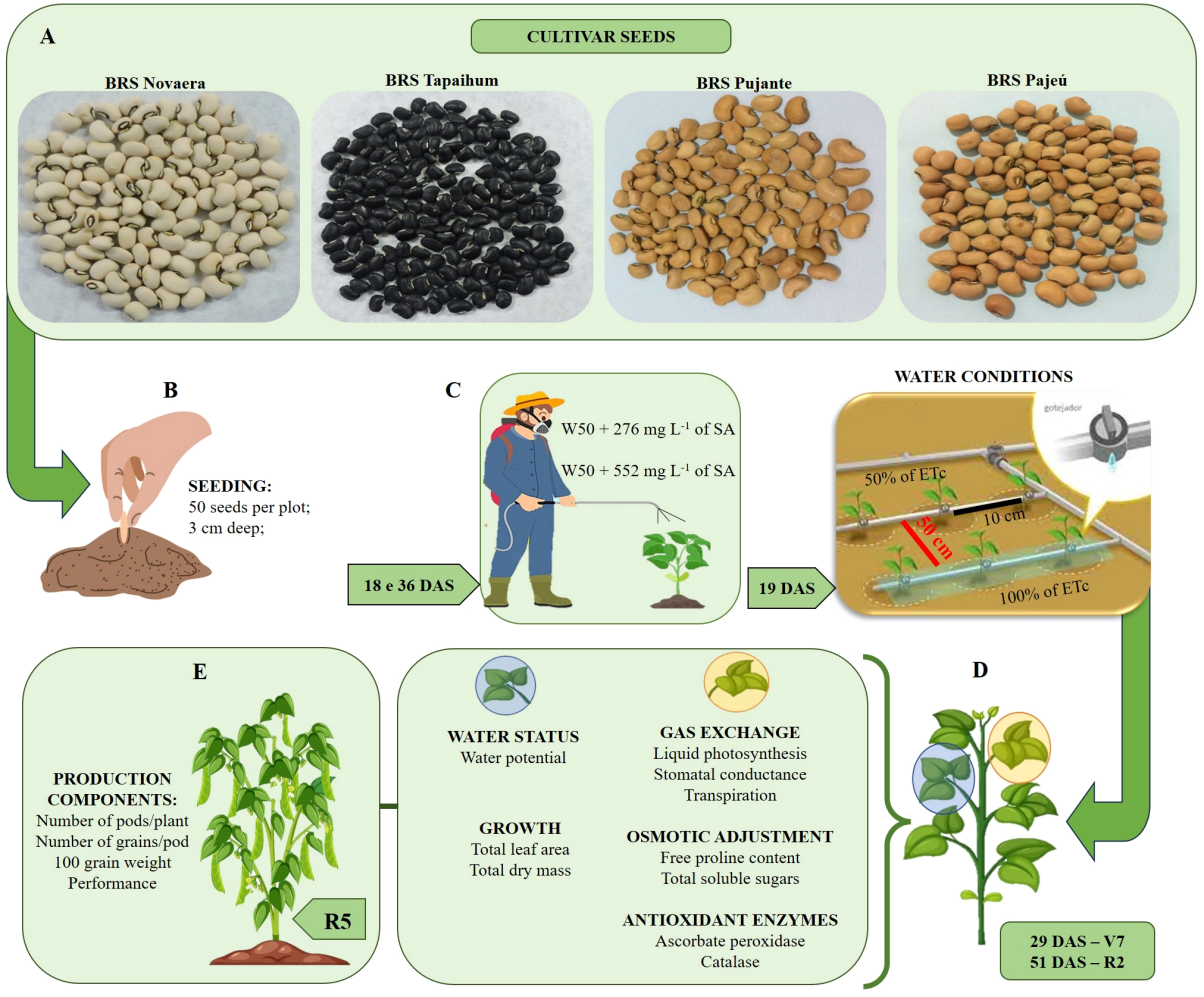
Experiment conducted and application of treatments. **(A)** cultivar seeds, **(B)** sowing, **(C)** application of SA concentrations at 18 and 36 days after sowing (DAS) and differentiation of irrigation depths (19 DAS), **(D)** variables analyzed in phenological stages V7 and R2, and **(E)** production variables analyzed at the R5 phenological stage.

Plant evaluations were conducted at the R2 stage (51 DAS) and at the end of the crop cycle (R5 stage). The following agronomic characteristics were evaluated: a) the number of pods per plant (NPP) (by dividing the total number of pods by the number of plants in the plot); b) the number of grains per pod (NGP) (by counting the number of grains of 10 pods per irrigation line - 50 per plot); c) the weight of one hundred grains in grams (WOHG); and e) the grain yield (GY) (kg ha^-1^). The experiment conduction in resume is show in the **Figure 2**.

The data were analyzed using ANOVA (F test at 5% probability) and Tukey’s mean comparison test (*P* ≤ 0.05) in SISVAR 5.6 software (Ferreira, 2019). Pearson correlation was performed on the variables using ggcorrplot package R software v. 4.2.3.

## 3 Results

### 3.1 Salicylic acid improves cowpea’s water status and photosynthetic performance under water restriction

**Figure 3:**
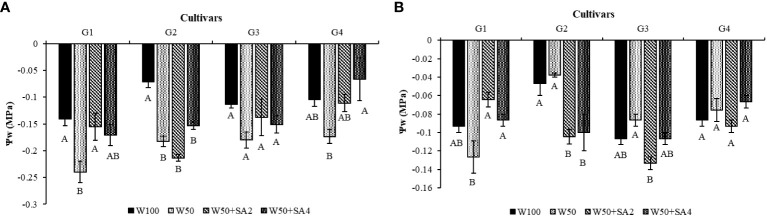
Leaf water potential (Ψw) in the phenological stages V7 **(A)** and R2 **(B)** of cowpea cultivars: BRS Novaera (G1), BRS Tapaihum (G2), BRS Pujante (G3), and BRS Pajeú (G4), subjected to control (W100), stress (W50), stress + 276 mg L^-1^ of SA (W50+SA2), and stress + 552 mg L^-1^ of SA (W50+SA4) treatments. Capital letters differentiate treatments within each cultivar (Tukey *P* ≤ 0.05), and lowercase letters differentiate cultivars within each treatment (Tukey *P* ≤ 0.05).

The study found that leaf water potential (Ψw) significantly decreased in the cultivars BRS Novaera (71.43%) and BRS Tapaihum (156%) under water restriction and absence of SA (W50) compared to W100 at stage V7 (**Figure 3A**). At the R2 phenological stage, in the same situation, a reduction was observed only in BRS Tapaihum (**Figure 3B**).

At the V7 phenological stage, even under stress conditions, applying 276 mg L^-1^ of SA to the BRS Novaera cultivar resulted in a 37.5% increase in leaf water potential, while applying 552 mg L^-1^ to BRS Pajeú resulted in a similar increase compared to W50 (**Figure 3A**). Notably, the plants treated with these concentrations had similar water potential to those receiving 100% water replacement (**Figure 3A**).

Water restriction significantly affected the gas exchange parameters evaluated in this study. However, the application of SA in some cultivars avoided these effects by maintaining the evaluated parameters in plants under stress at levels similar to those subjected to 100% water replacement.

**Figure 4:**
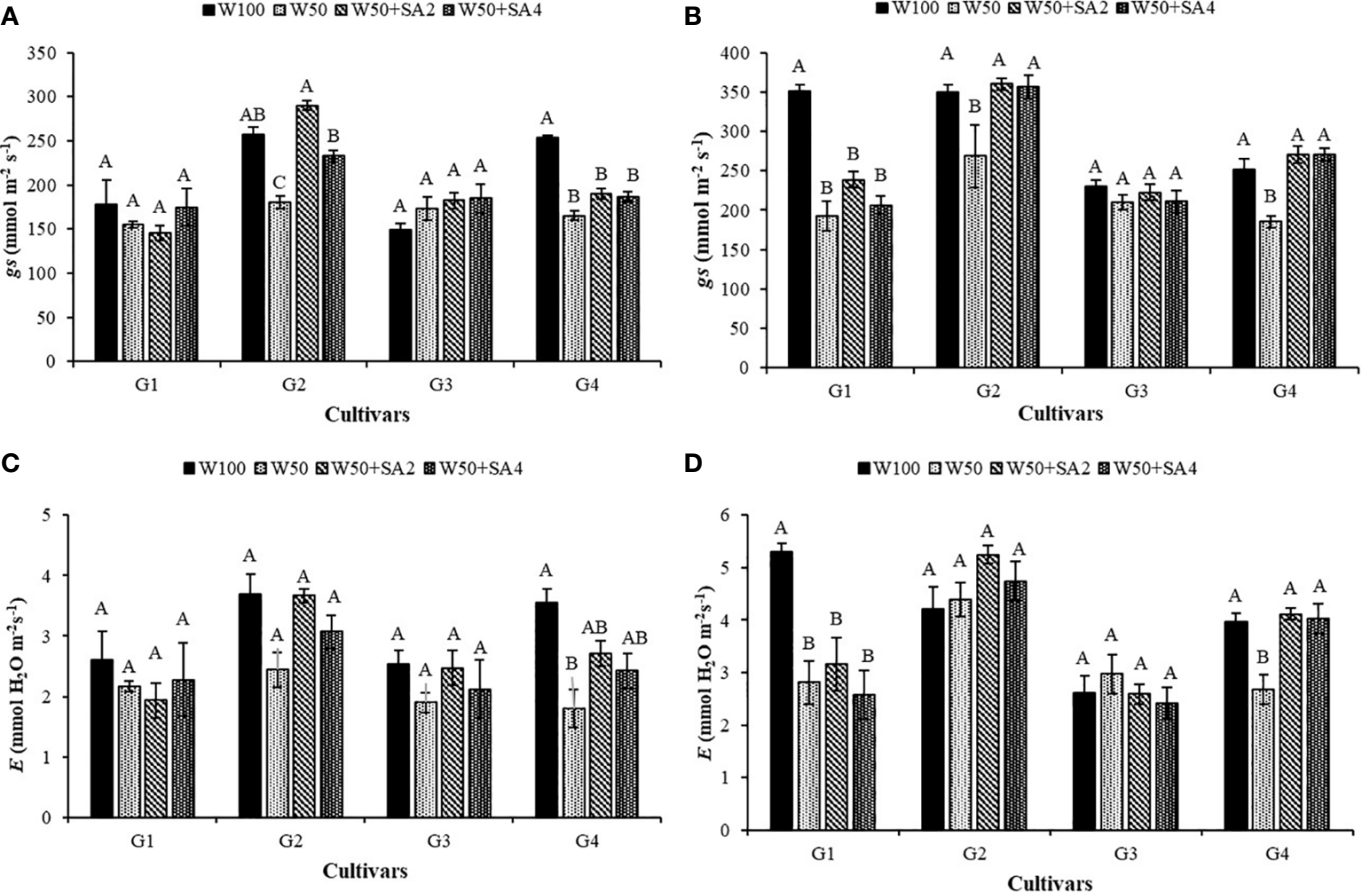
Stomatal conductance (*gs*) in phenological stages V7 **(A)** and R2 **(B)**, and transpiration (*E*) in stages V7 **(C)** and R2 **(D)** of cowpea cultivars: BRS Novaera (G1), BRS Tapaihum (G2), BRS Pujante (G3), and BRS Pajeú (G4), subjected to treatments control (W100), stress (W50), stress + 276 mg L^-1^ of SA (W50+SA2), and stress + 552 mg L^-1^ of SA (W50+SA4). Capital letters differentiate treatments within each cultivar (Tukey *P* ≤ 0.05), and lowercase letters differentiate cultivars within each treatment (Tukey *P* ≤ 0.05).

In the absence of SA, water restriction reduced stomatal conductance (*gs*) and transpiration (*E*) of the BRS Tapaihum and BRS Pajeú cultivars compared to the control treatment (W100) at stage V7 (**Figure 4A**). Under stress conditions, applying 276 mg L^-1^ of SA increases *gs* by 68% and *E* by 50% in the BRS Tapaihum cultivar, while with 552 mg L^-1^, it increases by 29% compared to W50 (**Figure 4A**).

At stage R2, the BRS Novaera cultivar exhibits a decrease in *gs* at 50% of the crop evapotranspiration (ETc) regardless of the treatment, compared to the W100 level. This behavior is also observed in the transpiration values of this cultivar (**Figures 4B, D**). Meanwhile, under stress, the cultivars BRS Tapaihum and BRS Pajeú only show a reduction in *gs* in plants that did not receive SA. However, this effect is reversed with the application of SA (treatments W50+SA2 and W50+SA4), where *gs* is significantly higher than in treatment W50 and equal to that observed in W100 (**Figure 4B**). It can also be highlighted that the BRS Pajeú cultivar presents similar behavior for *E*, which was reduced under water stress and in the absence of SA, as well as was significantly higher than plants subjected to SA application in the same water condition and at both concentrations (**Figure 4D**).

**Figure 5:**
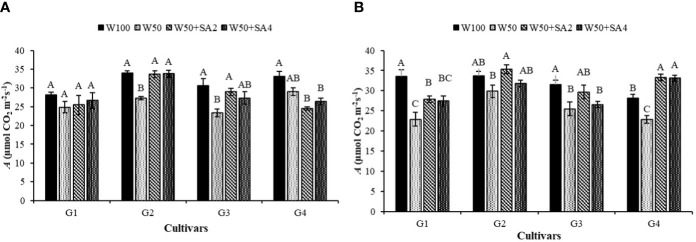
Photosynthesis (*A*) in the phenological stages V7 **(A)** and R2 **(B)** of cowpea cultivars: BRS Novaera (G1), BRS Tapaihum (G2), BRS Pujante (G3), and BRS Pajeú (G4), subjected to control (W100), stress (W50), stress + 276 mg L^-1^ of SA (W50+SA2), and stress + 552 mg L^-1^ of SA (W50+SA4) treatments. Capital letters differentiate treatments within each cultivar (Tukey *P* ≤ 0.05), and lowercase letters differentiate cultivars within each treatment (Tukey *P* ≤ 0.05).

In **Figure 5A**, for photosynthesis (*A*), at the V7 stage and in the W50 treatment, there is a 19% reduction in the BRS Tapaihum cultivar and a 23% reduction in the BRS Pujante cultivar compared with the control (W100). However, the application of SA (276 mg L^-1^) reversed the negative effect of water restriction on *A* for these cultivars. The plants subjected to the W50+SA2 treatment showed a higher photosynthetic rate than those in the W50 treatment and were statistically similar to the W100 treatment.

The cultivar BRS Tapaihum, exhibited a positive response to SA at a concentration of 552 mg L^-1^. However, the cultivar BRS Pujante did not show a significant difference when compared to the W50 treatment at this concentration. BRS Pajeú, subjected to SA application, shows a significant reduction in *A* compared to the W100 treatment, while the W50 treatment does not differ statistically from this one (**Figure 5A**).

At the R2 stage and in the W50 treatment, a decrease in *A* was observed in the cultivars BRS Novaera, BRS Pujante, and BRS Pajeú, compared to W100 (**Figure 5B**). It is important to note that, except for the BRS Pujante cultivar, all other cultivars showed a reduction in *gs* at this stage, which may have contributed to the decrease in *A* observed.

At this phenological stage, the beneficial effect of SA can be verified by the increases in *A* observed under stress conditions and at the concentration of 276 mg L^-1^ for the cultivars BRS Novaera, BRS Tapaihum, and BRS Pujante. Further, with both concentrations, BRS Pajeú showed higher average *A* values than those observed in plants that did not receive this attenuator under the same water condition (**Figure 5B**).

### 3.2 Indicators of osmotic adjustment and the antioxidant mechanism of cowpea under water restriction are enhanced with salicylic acid

**Figure 6:**
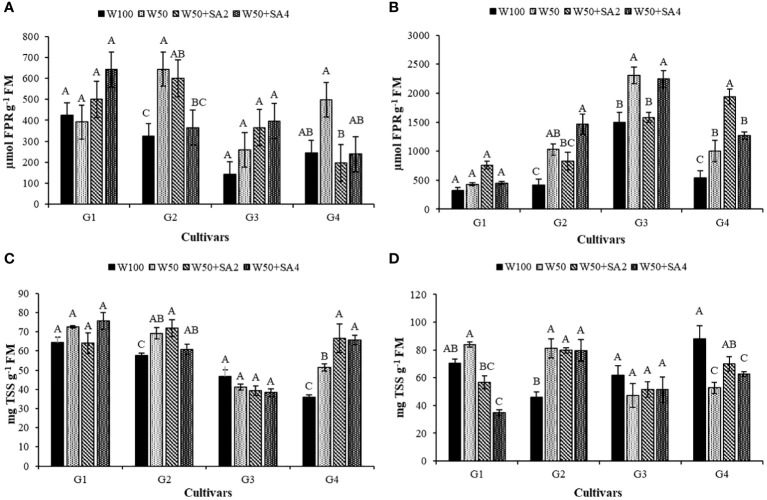
Free proline (FPR) content in phenological stages V7 **(A)** and R2 **(B)**; and total soluble sugars (TSS) in the V7 **(C)** and R2 **(D)** stages of cowpea cultivars: BRS Novaera (G1), BRS Tapaihum (G2), BRS Pujante (G3), and BRS Pajeú (G4), subjected to control (W100), stress (W50), stress + 276 mg L^-1^ of SA (W50+SA2), and stress + 552 mg L^-1^ of SA (W50+SA4) treatments. Capital letters differentiate treatments within each cultivar (Tukey *P* ≤ 0.05), and lowercase letters differentiate cultivars within each treatment (Tukey *P* ≤ 0.05).

The BRS Tapaihum and BRS Pajeú cultivars showed an increase in the content of free proline (FPR) under water restriction and absence of SA in both phenological stages (**Figures 6A, B**) and in total soluble sugars (TSS) in the V7 stage (**Figure 6C**), with values higher than those observed in the W100 treatment. The FPR of the BRS Novaera cultivar did not differ between irrigation depths without SA treatment. The BRS Pajeú cultivar showed an increase in FPR at W50, only at the R2 stage (**Figures 6A, B**).

The BRS Tapaihum cultivar showed a 63% reduction in FPR with the 552 mg L^-1^ SA treatment compared to the W50 treatment. The BRS Pajeú cultivar had reductions of 60% and 52% with concentrations of 276 and 552 mg L^-1^ at stage V7, respectively (**Figure 6A**), and increased TSS content with both concentrations at the same phenological stage (**Figure 6D**). At stage R2 (**Figures 6B, C**), the BRS Tapaihum cultivar shows an increase in FPR corresponding to 42% in relation to the W50 treatment. At a concentration of 552 mg L^-1^ of SA, it maintained TSS levels similar to W50 with both SA concentrations. Meanwhile, the BRS Pajeú cultivar exhibits a 93% increase in FPR with 276 mg L^-1^ (**Figure 6B**) compared to the W50 treatment.

**Figure 7:**
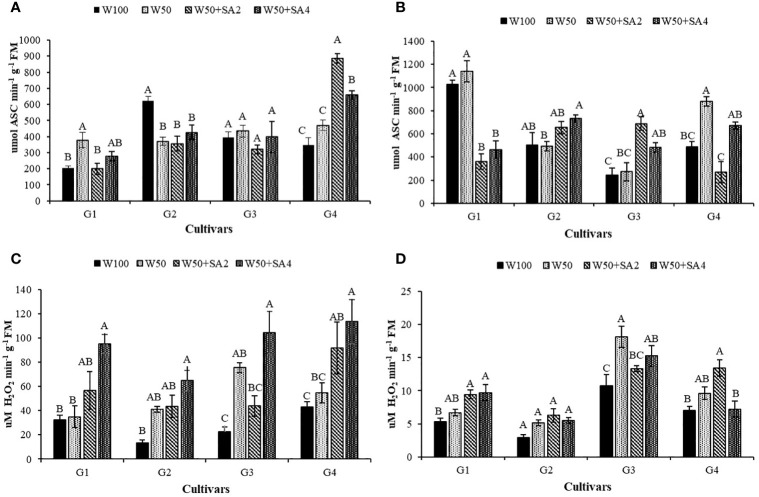
Activity of the enzyme ascorbate peroxidase (APX) in the phenological stages V7 **(A)** and R2 **(B)** and catalase (CAT) in the stages V7 **(C)** and R2 **(D)** of cowpea cultivars: BRS Novaera (G1), BRS Tapaihum (G2), BRS Pujante (G3), and BRS Pajeú (G4), subjected to treatments control (W100), stress (W50), stress + 276 mg L^-1^ of SA (W50+SA2) and stress + 552 mg L^-1^ of SA (W50+SA4). Capital letters differentiate treatments within each cultivar (Tukey *P* ≤ 0.05) and lowercase letters differentiate cultivars within each treatment (Tukey *P* ≤ 0.05).

Under water restriction and without the application of SA, the BRS Novaera cultivar showed an 84.7% increase in APX activity compared to the control treatment (W100), while the cultivar BRS Pajeú exhibited a 231% increase in catalase enzyme (CAT) activity under the same condition and phenological stage (**Figures 7A, C**). Additionally, when plants are under inadequate irrigation, applying SA increases the activity of these enzymes, suggesting that this acid has a positive effect in reducing the impact of water restriction at this phenological stage. The BRS Pajeú cultivar showed an 88% increase in APX activity with a concentration of 276 mg L^-1^ and a variation between 172% and 107% in CAT activity, with 552 mg L^-1^ of SA in the BRS Novaera and BRS Pajeú cultivars, respectively (**Figures 7A, C**).

At stage R2, when water was restricted and SA was absent, only the cultivar BRS Pajeú showed an increase in APX activity, which was 80% higher than that observed in W100 (**Figure 7B**). As for the catalase enzyme, this behavior was only observed in the cultivar BRS Pujante (**Figure 7D**). At this stage, the BRS Novaera cultivar has reduced APX activity at both SA concentrations, while the BRS Pajeú cultivar shows reduced enzyme activity with the application of 276 mg L^-1^. However, the BRS Pujante cultivar shows 155% and 78% higher activity than that observed in W50, with concentrations of 276 and 552 mg L^-1^ of SA, respectively (**Figure 7B**). Regarding the CAT enzyme, a positive effect of SA is observed in the cultivars BRS Novaera and BRS Paje under stress conditions in treatments W50+SA4 and W50+SA2, respectively, which supports the beneficial effect of SA in inducing resistance to water stress (**Figure 7D**).

**Figure 8:**
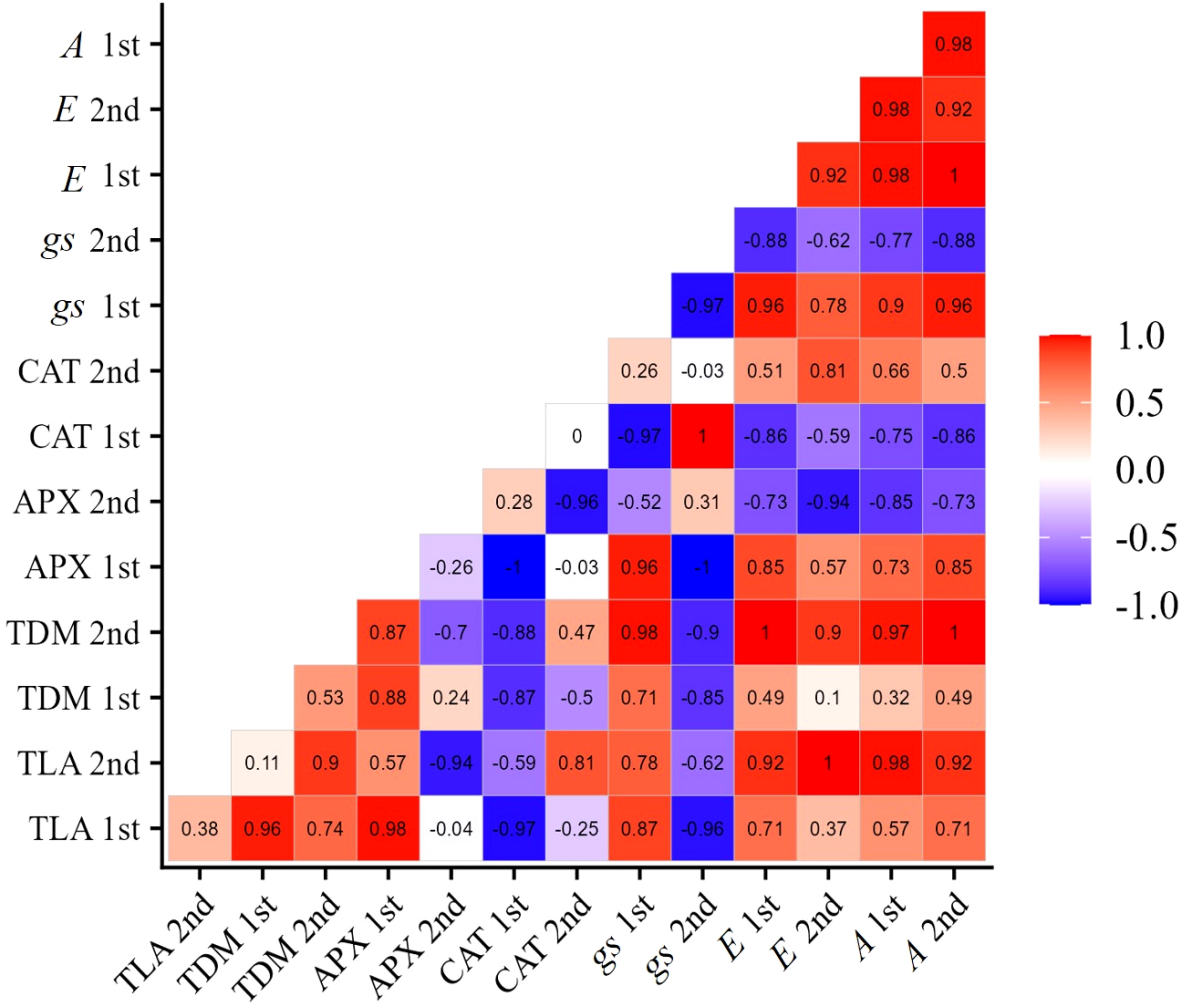
Correlation matrix of variables: net photosynthesis (*A*), transpiration (*E*), stomatal conductance (*gs*), catalase (CAT), ascorbate peroxidase (APX), total dry mass (TDM), and total leaf area (TLA), of cowpea cultivar BRS Pajeú, under water restriction (50% of ETc) and application of 276 mg L^-1^ of SA, in phenological stages V7 (1st) and R2 (2nd).

In cultivar BRS Pajeú, the APX enzyme activity is positively correlated with net photosynthesis and growth variables at the V7 stage. Similarly, at stage R2, these variables also show a positive correlation with CAT activity. These results suggest that exposing plants to the concentration of 276 mg L^-1^ of SA in the early stages of stress can increase APX activity and allow for an improvement in net photosynthesis, which may have a positive impact on the growth of these cultivars. In the subsequent moments, this fact is ensured by the CAT enzyme, which has its activity increased in the R2 stage (**Figure 8**), positively correlating with photosynthesis and plant growth in this same phenological stage. These results demonstrate the significance of the activity of these enzymes in tolerating the damage caused by water stress, as well as elucidating the role of salicylic acid in this process.

### 3.3 Salicylic acid mitigates the effects of water deficit on the growth and production components of cowpea

**Figure 9:**
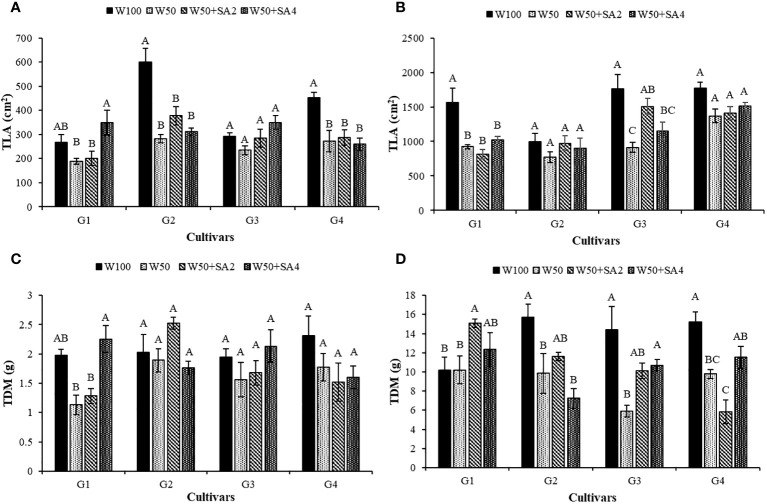
Total leaf area (TLA) at phenological stages V7 **(A)** and R2 **(B)**; and total dry mass (TDM) in stages V7 **(C)** and R2 **(D)** of cowpea cultivars: BRS Novaera (G1), BRS Tapaihum (G2), BRS Pujante (G3), and BRS Pajeú (G4), subjected to control (W100), stress (W50), stress + 276 mg L^-1^ of SA (W50+SA2) and stress + 552 mg L^-1^ of SA (W50+SA4) treatments. Capital letters differentiate treatments within each cultivar (Tukey *P* ≤ 0.05) and lowercase letters differentiate cultivars within each treatment (Tukey *P* ≤ 0.05).

At stage V7, water restriction negatively affected the total leaf area (TLA) of BRS Tapaihum and BRS Pajeú cultivars, regardless of SA application (**Figure 9A**). Meanwhile, at the R2 stage (**Figure 9B**), this behavior is observed in the cultivars BRS Novaera and BRS Pujante (W50 and W50+SA2).

At stage V7, a negative impact of water restriction was only seen in the BRS Novaera cultivar. However, this effect was reversed with the application of 552 mg L^-1^ of SA, leading to a 99.1% increase in total dry mass (TDM) (**Figure 9C**). At the R2 stage, a decrease in TDM was observed under water stress without SA in the cultivars BRS Tapaihum, BRS Pujante, and BRS Pajeú (**Figure 9D**).

**Figure 10:**
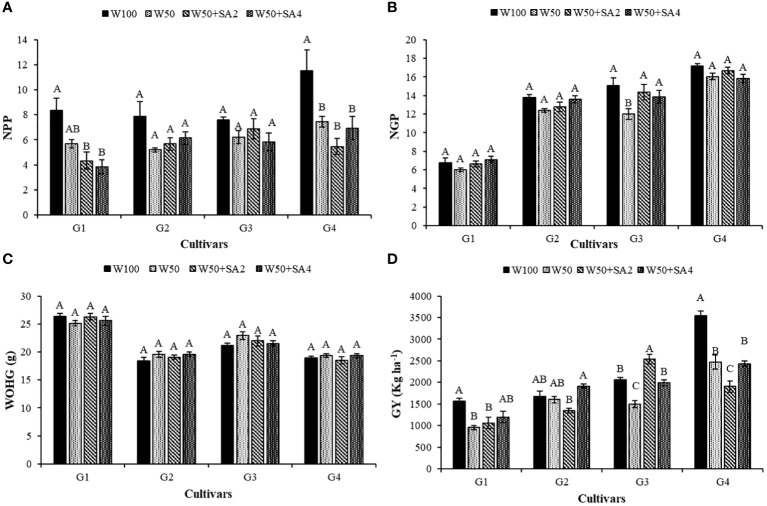
Number of pods per plant (NPP) **(A)**, number of grains per pod (NGP) **(B)**, weight of one hundred grains (WOHG) **(C)** and grain yield (GY) **(D)**, of cowpea cultivars: BRS Novaera (G1), BRS Tapaihum (G2), BRS Pujante (G3), and BRS Pajeú (G4) subjected to treatments control (W100), stress (W50), stress + 276 mg L^-1^ of SA (W50+SA2) and stress + 552 mg L^-1^ of SA (W50+SA4), at the R5 phenological stage. Capital letters differentiate treatments within each cultivar (Tukey *P* ≤ 0.05) and lowercase letters differentiate cultivars within each treatment (Tukey *P* ≤ 0.05).

Water deficit has a negative effect on the number of pods per plant (NPP) in the BRS Novaera and BRS Pajeú cultivars (**Figure 10A**). In contrast, the BRS Tapaihum and BRS Pujante cultivars showed greater resistance to water restriction, as they did not exhibit a difference in NPP between stressed plants and the control treatment (W100). The application of SA did not affect the NPP of tested cultivars because the plants subjected to the application of this attenuator presented values statistically similar to the W50 treatment (**Figure 10A**).

Water restriction and the absence of SA decreased the number of grains per pod (NGP) only in the BRS Pujante cultivar. However, applying concentrations of 276 and 552 mg L^-1^ of SA (**Figure 10B**) reversed this effect, increasing NGP by 19.7% and 15.5%, respectively, compared to plants that were not treated with this attenuator.

The productivity of BRS Novaera and BRS Pajeú was negatively affected by water stress, even with SA application. BRS Pujante cultivar showed a reduced productivity only in the W50 treatment under water stress (**Figure 10D**).

The BRS Pujante cultivar’s productivity decreased under water stress but increased by 69.3% with 276 mg L^-1^ of SA compared to the W50 treatment. It reached a productivity of 2,532.83 kg ha^-1^, the highest grain yield (GY) for this cultivar (**Figure 10D**). This cultivar treated with 552 mg L^-1^ of SA showed similar productivity (1,996.27 kg ha^-1^) to those receiving 100% water replacement. Productivity was 25% higher when compared to treatment W50 (1,496.20 kg ha^-1^) (**Figure 10D**), suggesting that SA can maintain productivity in this cultivar under water deficit conditions.

The BRS Pajeú cultivar showed the highest productivity among the cultivars tested (**Figure 10D**), even though its yield decreased with the W50 depth. This highlights the cultivar’s potential for high productivity in different conditions. BRS Tapaihum exhibited higher tolerance to water restriction in this study as its productivity did not differ between irrigation depths in the absence of SA.

**Figure 11:**
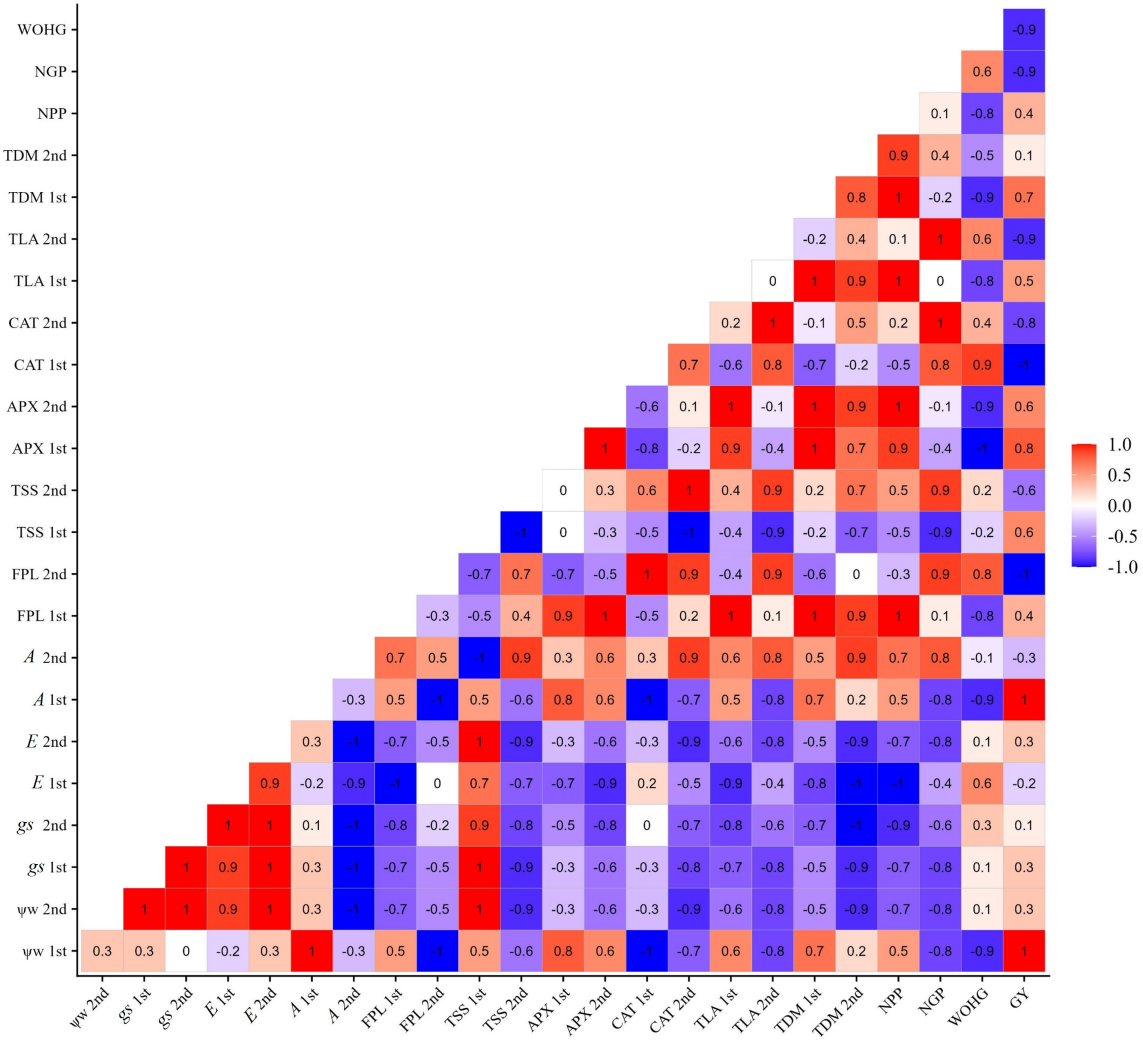
Correlation matrix of variables water potential (Ψw), total leaf area (TLA), total dry mass (TDM), free proline content (FPR), total soluble sugars (TSS), ascorbate peroxidase (APX), catalase (CAT), stomatal conductance (*gs*), transpiration (*E*), net photosynthesis (*A*), number of pods per plant (NPP), number of grains per pod (NGP), weight of one hundred grains (WOHG), and grain yield (cultivate bean- cowpea BRS Pujante), under water restriction (50% of ETc) and application of 276 mg L^-1^ of SA, in phenological stages V7 (1st) and R2 (2nd).

**Figure 11** shows that the increase in yield in the BRS Pujante cultivar presents a positive correlation with leaf water potential (1st and 2nd collections), ascorbate peroxidase enzyme activity (APX 1st and 2nd collections), net photosynthesis (1st and 2nd collections), and number of pods per plant. Additionally, it is possible to verify a positive correlation between leaf water potential and the content of osmotic adjusters (TSS and FPR) in the two phenological stages, which were also positively correlated with APX, *A*, and NPP.

## 4 Discussion

In the present study, the BRS Novaera and BRS Tapaihum cultivars showed a reduction in leaf water potential when subjected to water restriction. This restriction affected gas exchange and growth indicators, as evidenced by the reductions observed in stomatal conductance, transpiration (**Figure 4**), leaf area, and dry mass (**Figure 9**). Therefore, reducing leaf transpiration is a key strategy plants use to prevent dehydration and survive drought conditions (Ferreira et al., 2021). When faced with water scarcity, plants typically lower transpiration rates by decreasing leaf area (Kapoor et al., 2020) and/or closing stomata (Souza et al., 2020b), as demonstrated in this study. While this mechanism is an interesting survival strategy in drought conditions, Ferreira et al. (2021) point out that it can restrict the influx of CO_2_ and have a negative impact on photosynthesis. This report explains the reduction observed in net photosynthesis of the BRS Tapaihum and BRS Pajeú cultivars (**Figure 5A**).

It is important to note that a water deficit can lead to a decrease in chlorophyll content, which can inhibit photosynthesis (Parveen et al., 2021). This may have contributed to the observed reductions in BRS Pujante, as this cultivar did not exhibit a difference in stomatal conductance (*gs*) between different water conditions (**Figure 4A**).

Furthermore, under water restriction, it is also important to highlight that cowpeas can accumulate osmoprotective molecules, such as proline (Santos et al., 2022) and total soluble sugars (Jales Filho et al., 2023). These compounds favor water absorption, especially in low water availability soils, and are key mechanisms for plants to resist drought stress (Mohammadi et al., 2019; Santos et al., 2022; Gao et al., 2023; Jales Filho et al., 2023). In our study, we observed a similar response in the cultivars BRS Tapaihum, BRS Pujante, and BRS Pajeú. There were significant increases in FPR and TSS contents under water restriction (**Figure 6**).

Moura et al. (2016) reported that under water deficit conditions, the concentrations of TSS molecules change due to their impact on the translocation of photoassimilates in plants. This leads to a reduction in their utilization, causing them to accumulate as compatible organic solutes in the leaves. This accumulation contributes to the osmotic adjustment process (Coelho et al., 2018).

In plants under water restriction, applying SA can help increase leaf water potential to levels comparable to fully hydrated plants (**Figure 3**). This can help plants overcome the effects of water deficits by promoting stomatal conductance (**Figure 4**). These positive effects are linked to this elicitor’s role in promoting the production of osmoprotective molecules (Sultana et al., 2024). Jales Filho et al. (2023) observed that applying SA to cowpea cultivars under water restriction increased organic solute content and maintained water status in the plants, as verified in the present study (**Figures 6B–D**).

Therefore, despite changes in water potential, it is evident that SA mitigated the damaging effects of the water deficit. The adjustments in Ψw observed with the application of this substance can enhance the stomatal opening mechanism (Carvalho et al., 2020), leading to increased photosynthetic rates (**Figures 3B**).

During water restriction, some cultivars exhibited elevated activity of antioxidant enzymes in the assessed phenological stages (**Figure 7**). Santos et al. (2022) reported that, under water stress, the heightened activity of these enzymes serves as a crucial defense mechanism against oxidative stress, aiding in the preservation of cell membrane integrity (Ghahremani et al., 2023). However, the activity of antioxidant enzymes in response to water stress varies dep ending on the tolerance level of each cultivar (Carvalho et al., 2019). This explains the diverse responses observed in the evaluated cultivars. It is remarkable that SA can act as a non-enzymatic antioxidant, helping to remove ROS and regulate their levels in cellular metabolism (Arif et al., 2023). This could explain the decrease in APX enzyme activity in BRS Novaera and BRS Pajeú cultivars during the R2 stage, even under water restriction (**Figure 7B**).

Additionally, under water restriction, the application of SA increased the activity of antioxidant enzymes such as APX and CAT, potentially helping to alleviate the negative impacts of water stress in the studied cultivars (Shemi et al., 2021; Sultana et al., 2024). Similar findings were reported by Oliveira et al. (2023) in the cowpea cultivar BRS Pajeú, where SA application at a concentration of 0.21 g L^−1^ led to an increase in APX activity. Jales Filho et al. (2023) also observed a similar response in the cowpea cultivar BRS Paulistão, highlighting the beneficial effects of SA in eliciting responses to water stress.

Water deficits can promote a series of physiological and morphological changes in cowpea plants, depending on the intensity, phenological stage, and cultivar resistance (Melo et al., 2022). During the reproductive phase, a water deficit can result in flower and pod loss, decreasing the number of pods per plant (Barros et al., 2021); this effect was observed in the BRS Novaera and BRS Pajeú cultivars (**Figure 10A**). For the BRS Pujante cultivar, the water deficit reduced NPP and impacted the production of photoassimilates necessary for grain production (**Figure 10B**), this result is corroborated by Martins et al. (2017).

There was no effect of treatments on cowpea cultivars for the weight of one hundred grains (WOHG) (**Figure 10C**). This result, as explained by Locatelli et al. (2014), is likely due to the high genetic heritability of WOHG, making it more resistant to environmental factors. Valeriano et al. (2019) found that this variable is associated with the movement of photoassimilates within the plant and is influenced by the source/sink relationship. It is important to highlight that the average WOHG values of the BRS Novaera and BRS Pujante cultivars meet the preferences of producers, buyers, and packers, who typically prefer grains weighing over 20 g per 100 grains, as reported by Públio Júnior et al. (2017).

The results demonstrate that water deficits can negatively impact cowpea production, and these effects can be directly linked to reductions in gas exchange caused by water restriction, particularly affecting stomatal conductance (*gs*) (Souza et al., 2020b). Stomatal closure limits the assimilation of CO_2_ and affects the photosynthetic process, thereby impacting the production of photoassimilates necessary for grain formation (Ferreira et al., 2021).

In the BRS Pujante cultivar, the negative impact of the water deficit on productivity (**Figure 10D**) was mitigated by SA at a concentration of 272 mg L^-1^. This suggests that the SA treatment increased the levels of osmotic adjusters, helping to maintain optimal leaf water potential and boosting APX activity in both phenological stages (**Figure 11**). These factors likely helped sustain net photosynthesis, leading to an increase in NPP (**Figure 10A**) and ultimately boosting the yield of the cultivar, even in conditions of water scarcity. Recent studies on wheat (El-Sanatawy and Zedan, 2020) and pea (Soni et al., 2021) plants have also demonstrated enhanced productivity in plants treated with SA under water stress.

## 5 Conclusions

Water restriction negatively affected water status, growth, gas exchange, and production of the four cultivars, as well as promoted changes in antioxidant metabolism and osmolyte concentration. The application of SA enhanced the antioxidant activity and the synthesis of osmotic adjusters, mitigating the effects of water restriction in cowpea. The concentration of 276 mg L^-1^ was more effective in stage R2 and 552 mg L^-1^ in the stage V7.

The BRS Pujante cultivar has increased its productivity under water restriction with SA application, and BRS Tapaihum is the most resistant among the cultivars studied.

Our findings underscore the importance of using SA to mitigate the effects of water restriction on cowpea cultivation. These discoveries are crucial for the sustainability of cowpea production in regions susceptible to drought, which can contribute to food security. We further add that the adoption of new agricultural practices can enhance the resilience and productivity of cowpea as an essential and sustainable food source for vulnerable populations in various parts of the world.

## Data availability statement

The raw data supporting the conclusions of this article will be made available by the authors, without undue reservation.

## Author contributions

IC: Investigation, Methodology, Visualization, Data curation, Formal analysis, Writing – original draft. AM: Investigation, Methodology, Visualization, Conceptualization, Funding acquisition, Project administration, Resources, Supervision, Writing – review & editing. RF: Investigation, Methodology, Visualization, Writing – review & editing. RA: Investigation, Methodology, Visualization, Writing – review & editing. GD: Investigation, Methodology, Visualization, Writing – review & editing. PV: Investigation, Methodology, Visualization, Writing – review & editing. MR: Visualization, Writing – review & editing. AN: Visualization, Writing – review & editing. FS: Visualization, Writing – review & editing. CL: Visualization, Writing – review & editing. PV: Visualization, Writing – review & editing.
